# Intercultural communicative competence in higher education through telecollaboration: typology and development

**DOI:** 10.1007/s10639-023-11751-3

**Published:** 2023-04-01

**Authors:** Elba Gutiérrez-Santiuste, Maximiliano Ritacco-Real

**Affiliations:** 1 Department of Pedagogy, University of Granada, Granada, Spain; 2 Granada, Spain

**Keywords:** Intercultural communicative competence, Higher education, Telecollaboration

## Abstract

This study aims to analyse intercultural communicative competence, understood as the individual’s ability to effectively and appropriately develop communication and behaviour, when interacting in an intercultural context. In this study, the Behavioural, Affective and Cognitive Dimensions, and their sub–dimensions, are considered by using videoconferencing as a tool for telecollaboration in Higher Education. These sub–dimensions are observed according to their positive and negative orientation (facilitating or inhibiting). The objectives of the current study are to analyse the dimensions and sub–dimensions distribution, to assess the incidence of the typology of generic and specific topics, and to assess the over time communication evolution. Content analysis of communications between university peers was carried out and we undertook a percentage frequency index. The results show behavioural communications to be in the majority, followed by affective and, finally, cognitive communications. Communications with a negative aspect are almost absent from this study. MANOVA was performed to investigate differences between typologies of topics (generic/specific) in dimensions. This research founds statistically significant differences in Affective Dimension. ANOVAs were conducted to observe if there are differences in the development over time of Behavioural, Affective and Cognitive Dimensions of intercultural online communications. There was a significant effect over time in Affective and Behavioural Dimension. The present study finds expressions that show a positive attitude towards communication, as well as interest in and an effort to maintain it. We can conclude that, in Affective Dimension, where generic topics enhance communication, while educational topics inhibit it. However, a sustained evolution over time has not been found, rather a significant incidence depending on topic themes.

## Introduction

One of the roles of Higher Education is to prepare students for interaction with people from other cultures in order to allow them to participate actively in a diverse society. In addition, the expansion of globalisation requires students to communicate appropriately and effectively (Akdere et al., 2021), since ‘all societies in our contemporary world are the result of intercultural communication’ (Deardoff, 2020, p. ix). Intercultural educational communication using telecollaboration offers opportunities to develop a wide range of competences, while allowing a reflection on the students’ own experiences. However, according to Avgousti (2018), more research is needed on the pedagogical uses of less studied Web 2.0 technologies.

The intercultural communicative competence (ICC) implies, on the one hand, the individual’s understanding of the norms of their own culture and other cultures. On the other hand, ICC implies an understanding of how to use that knowledge for successful communication with people who do not share the same cultural background and for the effective building of bridges in situations of cultural diversity (Akdere et al., 2021; Toscu & Erten, 2020). The development of this competence will surely influence feelings, identity and patterns of thinking. The latter may be different from one culture to another, understanding that these are the structures which organise ways of acting, interacting, values and personal imaginaries. Thus, Lantz–Deaton and Goluveba (2020) point out that, in intercultural exchange, each individual interprets reality through the lens of their own personality and their own cultural group. In addition, the relationship between cultural knowledge and communication competencies can be considered indissoluble, since culture marks the norms and conventions of communication (Avgousti, 2018).

This study has an innovative character since it is carried out through the interrelated analysis between the topics dealt with in the videoconferences (VC)—generic (food, politic, holidays, …) or specific (educational)—and the use of the communications themselves, not through self–reports, of the intercultural meetings between students in pairs. This interrelationship has not been analysed by previous research and may facilitate understanding of how ICC evolves. This study focuses on analysing communications between culturally diverse pairs of students developed through VC as a tool for telecollaboration. Specifically, it examines the issue by posing the following questions:

- How intercultural communication is distributed considering behavioural, affective and cognitive dimensions?
- What typologies of intercultural online educational communications can be observed among university peers?
- How do intercultural online educational communications develop over time?

## Background

### ***Intercultural communicative competence***

Since the second half of the 20th century various models for understanding ICC have arisen which complement each other and broaden the view that we currently have of this competence. Likewise, there is a long history of intercultural competence (IC) assessment in a variety of contexts, including higher education institutions (Iseminger et al., 2020). In the scientific literature, conceptual diversity is observed in the relationship between the terms ICC and IC. For some authors, this relationship is established by considering IC as the ability to communicate effectively and appropriately in intercultural situations (Akdere et al., 2021; Chen & Gabrenya, 2021; Deardorff, 2020). Here, the IC is properly communication: ‘the intercultural competence or intercultural communicative competence typically include the attitudes, skills and knowledge required in appropriate communications when interacting across difference’ (Deardorff, 2020, p. 5). Also in this sense, the proposal of Swartz and Shrivastava (2021) can be placed, pointing out that the ICC involves the cognitive, affective and behavioural attributes to communicate effectively across various cultures. Another trend in the conceptualisation of the relationship between ICC and IC is that proposed by Lantz–Deaton and Golubeva (2020). These authors argue that the ICC implies communication between people; that is, the IC is a broader term in which the ICC could be placed as a component. The ICC is considered by these authors as an overly specific term, since it suggests a focus only on communication between people from different linguistic backgrounds. For Lantz-Deaton and Golubeva (2020), the base of IC is developed through ICC, which makes it possible to identify the development of IC through communications. In this case, the ICC is the tool to develop the IC. Assuming one or another conceptual trend, it can be considered, as Deardoff (2020) points out, that the ways of developing IC involve communication skills. This is because, in the end, communication and behaviour are at the centre of intercultural competencies. Starting from the definitions proposed by Chen and Young (2012) and Deardoff (2020), in this research, ICC is defined as the individual skill required to develop in an effective and appropriate way a communication and behaviour when interacting in an intercultural context.

Moreover, the ICC is a multidimensional construction (Griffith et al., 2016; Lanz–Deaton & Golubeva, 2020) that includes, following the proposals of Akdere et al. (2021) and Lee and Song (2019), some combination of attitude and affect, knowledge/cognition, and skills/behaviour. The multidimensional aspects of the ICC show the complexity of its structure. These three dimensions—attitude, affect and behaviour—are the object of analysis in this study.

Following Heggernes (2021) and Deardorff (2020), the intercultural communication is essentially developed through a dialogue requiring a respectful atmosphere and sincere interest in the perspectives of others. Similarly, it can be considered that the ‘interpersonal interaction—conversational, institutional, task-based, digital, and so on—is situated; that is, it takes place in a particular sociocultural setting and therefore inherently involves issues of face’ (Van Der Zwaard & Bannink, 2020, p. 59). In addition, the IC is a set of related competencies that can be improved over time and as a result of experience. This is opposed to the idea of a collection of static personality traits; that is, intercultural competence is eminently learnable (Akdere et al., 2021).

Furthermore, the organisation of the underlying dimensions in the ICC under attitudes, behaviours and cognition is a structure used in previous research. In this way, the questionnaires analysed by Chen and Gabrenya (2021)—SCAS (Sociocultural Adaptation Scale, Ward & Kennedy, 1999); CCAI (Cross-Cultural Adaptation Inventory, Kelley & Meyers, 1995); MPQ (Multicultural Personality Questionnaire, Van der Zee & Van Oudenhoven, 2000); ISS (Intercultural Sensitivity Scale, Chen & Starosta, 2000); CQS (Cultural Intelligence Scales, Ang et al., 2007)—to examine the ICC match this structure (see Chen & Gabrenya, 2021, Fig. 1, p. 38). Their research suggests that, empirically, these instruments represent fewer dimensions than those originally stated by their developers, although all of them, among other dimensions, coincide in the three indicated above (attitudes, behaviours and cognition).

**Fig. 1:**
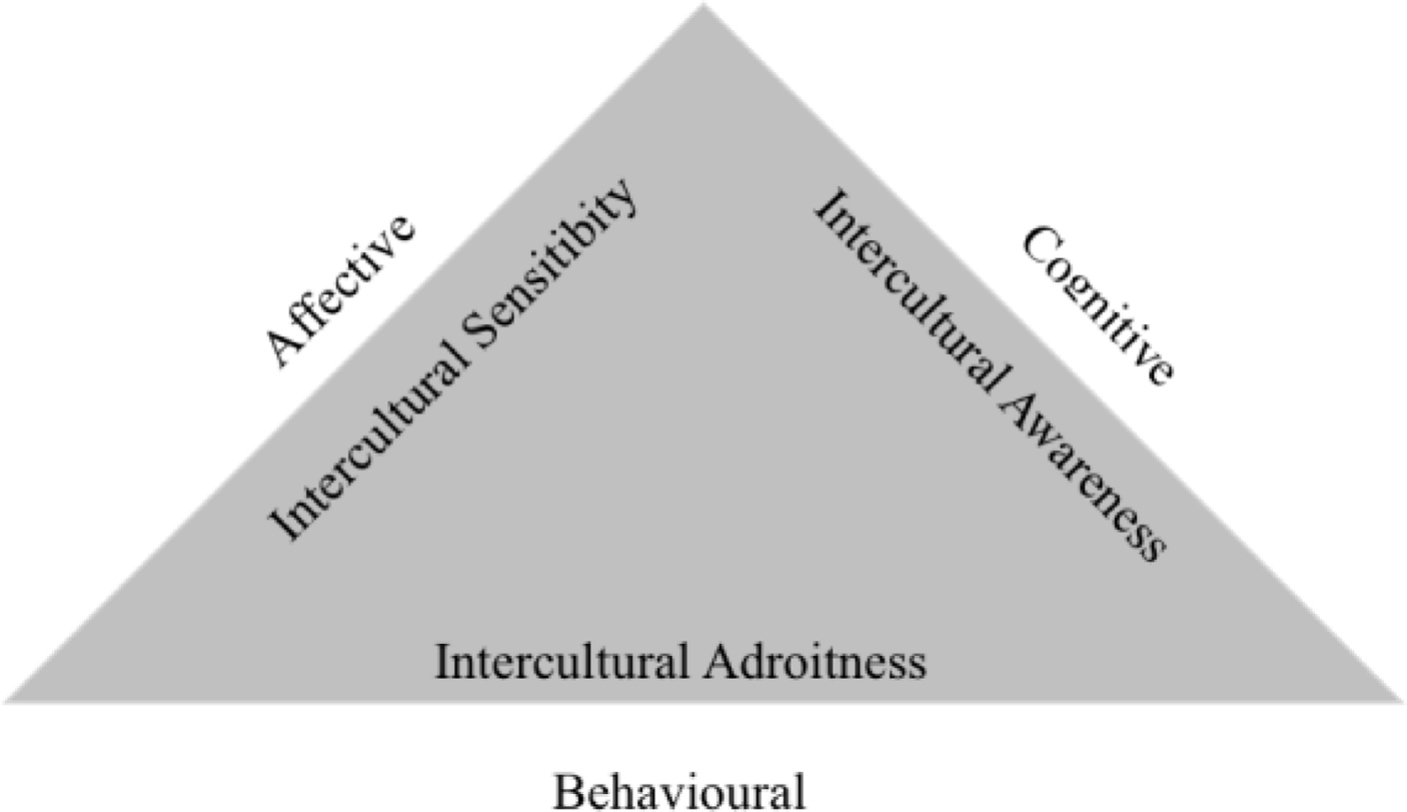
Model of intercultural communication competence (Chen & Young, 2012, p. 177).

This model is split into three elements which make up ICC: behavioural (intercultural adroitness), affective (intercultural sensitivity) and cognitive (intercultural awareness) (Fig. 1).

Lee and Song (2019) point out that so far behavioural elements constitute the least researched dimension, and define this element ‘as students’ willingness to learn about the target culture or their directed efforts to engage in behavior aimed at intercultural understanding’ (p. 180). According to Chen and Young (2012), intercultural adroitness ‘refers to the ability to achieve one’s communication goals in intercultural interaction through behavioural performance’ (p. 179). Verbal and non-verbal communication skills enable the individual to be successful, and productive, when engaged in the communication process.

With regard to behavioural elements, a number of skills are necessary for successful communication (Akdere et al., 2021; Chen & Young, 2012; Lee & Song, 2019; Swartz & Shrivastava, 2021; Toscu & Erten, 2020). They are as follows:

- Message skills: ability to employ verbal and nonverbal behaviours. Adaptation of behaviour to facilitate a successful interaction.
- Interaction management: ability to initiate, take turns, terminate a conversation.
- Behavioural flexibility: ability to take note of various information and to use appropriate communication strategies.
- Identity management: ability to maintain the personal and cultural identities of the partner.
- Conation: personal energy that has both direction (positive/negative) and magnitude (greater/lesser), connected to the will that indicates a behaviour.

The affective elements (Chen & Starosta, 2000) include intercultural sensitivity, which refers to the promotion of positive feelings and emotions during intercultural communication. According to Chen and Starosta (1997), these affective elements include ‘willingness or motivation to understand, acknowledge, respect, and even accept differences of the two cultural beings or groups’ (p. 231). Affective elements, according to Akdere et al. (2021), Chen and Starosta (2000), Griffith et al. (2016), Lee and Song (2019) and Toscu and Erten (2020) refer to:

- Cultural self-efficacy: when an individual develops close relationships with people from other cultures.
- Positive cultural orientation: cosmopolitanism (reduced ethnocentrism), open-mindedness, inquisitiveness and curiosity, which are complementary skills, willingness to learn from other cultures. Mutual respect for others also figures here (extent to which participants understand, accept and respect cultural differences). Individuals with this ability respect the differences between people from other cultures.
- Tolerance of ambiguity: intercultural interactions are, by nature, ambiguous. Individuals who tolerate ambiguity can continue to function/communicate despite the fact that the behaviours they observe differ from their own. Individuals with this ability have a high level of tolerance to stress.
- Interaction confidence: degree of trust felt by interlocutors during intercultural communication.
- Interaction enjoyment: level of pleasure that the interlocutors obtain from the communication.
- Interactional attentiveness: ability to respond observantly in communicative situations.

Finally, cognitive elements indicate intercultural awareness. These elements relate to understanding the characteristics of the target culture, its beliefs and values as well as the ways in which people from different cultures think (Chen & Starosta, 1997; Lee & Song, 2019). Following the arguments of Chen and Young (2012), on the one hand, it is necessary to reduce ambiguity and uncertainty (discomfort, confusion, anxiety) in order to develop the cognitive elements. These elements are also related to the appreciation of the differences between an individual’s culture of origin and other cultures (Lee & Song, 2019).

Other aspects of the cognitive elements are (Chen & Young, 2012; Toscu & Erten, 2020):

- Social monitoring: the ability to infer social norms, hierarchies and networks of interpersonal relationships.
- Suspending judgement and perspective taking: complementary skills which facilitate understanding situations without using strong personal bias, stereotypes or generalisations; skills of interpreting and relating.
- Cultural knowledge application: this skill refers to the use of cultural information in assessing and making decisions on how to act. Cultural information can include cultural values, specific cultural norms (for example how to greet others), historical and geopolitical information (e.g., trends of power and privilege), knowledge of one’s own culture.

### ***Educational videoconferences as tools for telecollaboration***

Telecollaboration is understood as an online intercultural exchange between people from different cultural backgrounds and is established through structured tasks in an institutional context (Godwin–Jones, 2019). For a more nuanced understanding of cultural influences, and following the work of this author, it would be useful to see each telecollaborative project as a manifestation of culture itself, a small culture, a project based on a common purpose, a shared environment, and an open form of communication. On the other hand, in virtual environments, learning about culture is experiential (Lee & Song, 2019); it is also subjective and not factual or objective (Avgousti, 2018). This conception defines culture as fluid and negotiated, rather than fixed and inherited (Liu & Shirley, 2021; Auvgousti, 2018). Lee and Song (2019) compared different learning scenarios and found that the group of learners who studied through telecollaboration, showed a significant improvement in all three main dimensions of ICC (affective, cognitive and behavioural). Toscu and Erten’s (2020) study also concludes that, in the group that used telecollaboration, the participants’ enthusiasm for learning about other cultures also improved. This did not occur in the other group. Chen and Yang (2016) report that telecollaboration helps participants to improve their affective states, including showing interest, curiosity and intrinsic motivation.

A tool used for telecollaboration is VC. Direct, visual and auditory contact between interlocutors can be highly motivating and allows learners to connect easily through their mobile devices or home computers. Recent evidence suggests that, due to its interactive and collaborative nature, VC is suitable for a more dynamic and responsive form of reflection, instilling trust and closeness, as well as greater depth and breadth in thinking (Dai, 2019; Lenkaitis et al., 2019), as long as the participants have enough bandwidth and video quality. In this sense, the findings of the study by Eren (2021) also suggest that VCs had a significant impact on the critical intercultural development of students. Specifically, the interaction with different cultural perspectives dismantled prejudices and stereotypes, facilitated the recognition of diversity, the negotiation of meaning, the creation of awareness about the relativity of cultural beliefs and the development of a pragmatic cultural stance.

Lee and Song (2019), using voice calls together with text messages and mixed methodology for their analysis, measured cognitive, affective and behavioural aspects. The results indicate that, over time, the group significantly improved in the cognitive, affective (engagement and trust), and behavioural aspects of ICC. On the other hand, Dai’s (2019) study concludes that videoconferencing can facilitate a deep level of cultural knowledge depending on the instructional design, thus creating a *glocal* learning experience. The study by Gómez-García, Gutiérrez-Santiuste and Moreno–López (2021) analyses intercultural communication under the model of Community of Inquiry, finding a large amount of social communication, above cognitive and teaching communications.

Traditionally, VC was considered a tool that did not facilitate student reflection due to its synchronous nature, since it generated pressure by having to offer an immediate response. Van der Zwaard and Bannink (2019) find that video exchanges sometimes hinder task performance because the presence of a webcam proves to be face-threatening. Videoconferencing can be demanding for participants as they must pay attention to, and interpret, the meaning of verbal expressions, as well as paralanguage, facial expressions and gestures. In addition, VC requires thinking and responding quickly as well as articulating effectively. Several studies have analysed negative stereotypes relating to the target culture. While some studies point to the reinforcement of cultural stereotypes (Flowers et al., 2019), other studies indicate the reverse (Eren, 2021), modifying attitudes and preconceptions shaped by the media. Furthermore, as pointed out by Custer (2017), there are potential dangers to learning curves because of the use of unfamiliar technology, and a learning management system that is known to one group of students but not to the other, or from technical or connection problems, particularly during synchronous exchanges.

In addition, following Eren (2021) and Godwin–Jones (2019), ‘safe’ or generic topics, such as food, music and travel, are valid starting points for an intercultural learning proposal, but they do not reflect a critical introspection of culture. According to Yasin (2016), VC is a tool which is used in digital–based intercultural learning with average frequency, although the Covid–19 pandemic has exponentially increased its use in education (Brooks, 2021; Farnell et al., 2021; Toscu & Erten, 2020) observed its positive influence on fluency and overcoming apprehension in intercultural communications, although they saw no effect on the attitudinal dimension of the participants. In this sense, our proposal is based on the students’ own communications and covers cognitive, affective and behavioural aspects taking, as a theoretical basis, Chen and Young’s (2012) proposal (intercultural awareness, intercultural sensitivity and intercultural behaviour).

On the other hand, Griffith et al. (2016) and Chen and Gabrenya (2021) note that there is little consensus on the measurement of ICC. Studies have relied too heavily on self–report methods which do not adequately cover the full spectrum of the construct. According to Chen and Gabrenya (2021, p. 51) ‘a problem shared [… by] self–reported instruments is reliance on knowledge, skills, attitudes, and behaviors, providing evidence for test–takers’ self-perceptions instead of their actual competence or performance’. Specifically, existing measures often exploit self-reference to cognitive aspects without adequately capturing the affective and behavioural aspects inherent in intercultural interactions. Tuscu and Erten (2020) have developed their research using mixed methods, concluding that videoconferencing is useful for the development of ICC.

In light of the literature analysed, it is considered that there is a gap in the knowledge of the evolution over time of synchronous communications through VCs; specifically, depending on the topics covered (whether generic or specific), in carrying out analysis of VCs through the communications themselves and not through self–reports.

## Methodology

The methodology of this study, content analysis, has been designed with the aim of analysing and interpreting the virtual communications between students from different places and cultures. The information has additionally been structured in order to obtain frequencies (counts and percentages). The use of VCs for the development of interculturality has been analysed from different methodological perspectives. An example of quantitative analysis is the study of Flowers et al. (2019). The most frequent analyses are those of mixed methodology. Among them, the study by Eren (2021) uses the questionnaire and the constant comparative method based on the Grounded Theory. Among the qualitative studies are those of Van Der Zwaard (2020), which uses content analysis; the study by Dai (2019), which is based on discourse analysis with an ethnographic approach; and the study by Lee and Song (2019), which, through qualitative methodology, analyses interviews, essays, and reflective writing.

**Fig. 2:**
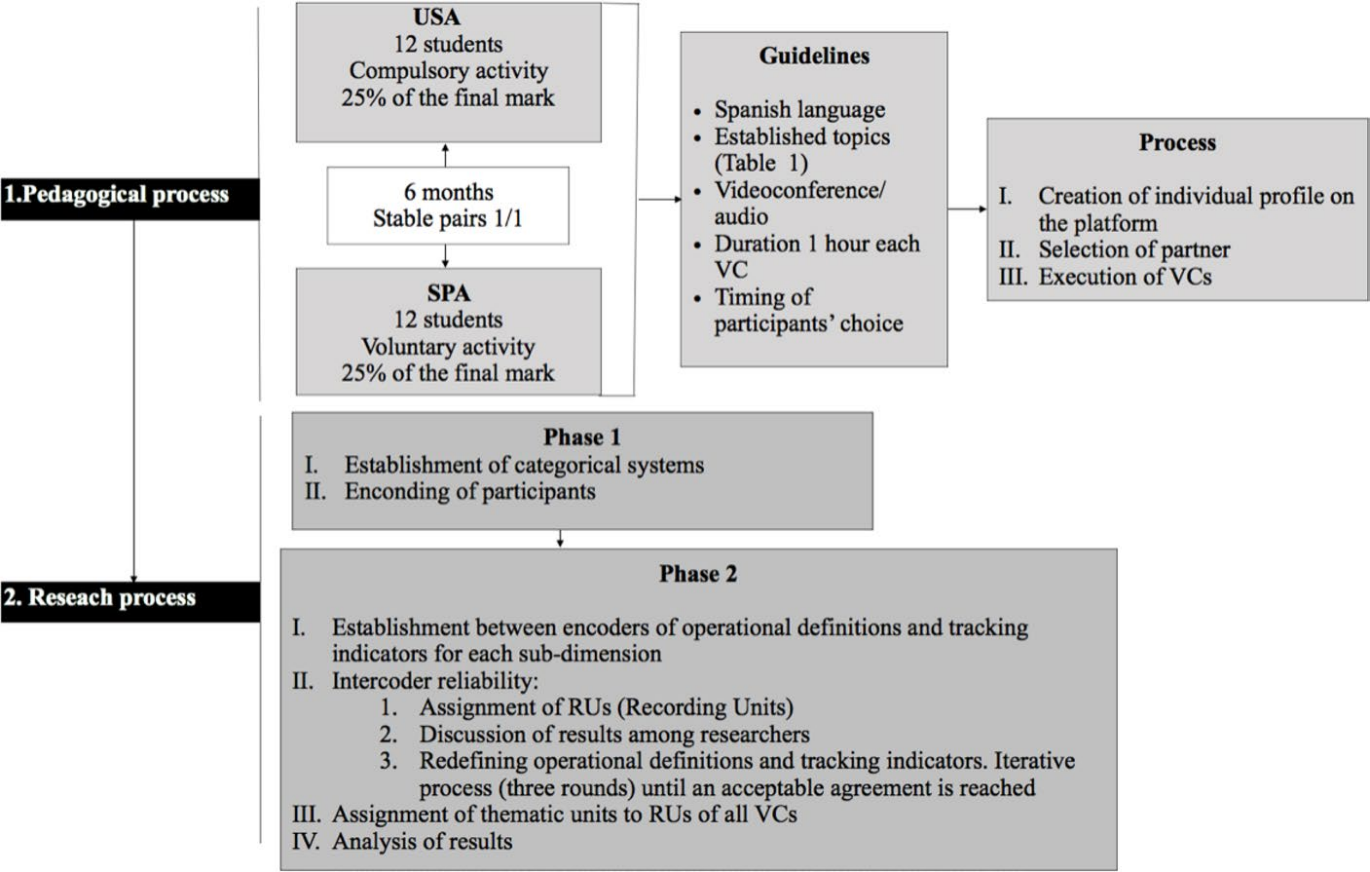
Experimental design.

Figure 2 shows the design and process used in this study.

### ***Pedagogical context of the experience***

This study analyses VCs exchanges between Spanish and American university students which took place over a period of six months. The contents of these VCs were organised on the basis of the learning objectives of two subjects:

- For the American students, the subject was Spanish language, which was taught five hours per week and involved three face–to–face and two compulsory online lessons.
- For the Spanish students, the subject was educational systems in primary education, which involved four face–to–face and two online hours of learning. For both groups, the VCs accounted for 25% of their final mark.

Due to the fact that the initial group of Spanish students was larger than the American group, two activities were chosen, one of them being the VCs. The first 12 students who expressed an interest in this activity formed the VCs group.

Some guidelines for all students were established beforehand. Their aim was: to create an individual online profile and share it with their peers on an educational platform; to allow each pair of students to choose their preferred communication tool in order to develop the VCs; to prioritise the use of the Spanish language but, if necessary, to allow the use of English; to prioritise the pre–established topics of conversation (Table 1), with the possibility of addressing other emerging topics of interest; to record the VC in both audio and video. In the event of technical problems an audio recording would be sufficient, for each couple to set their own schedule of meetings, each lasting approximately one hour.

**Table 1:** Conversation sequence and script for the VCs

Sequence	Title	Topics for the conversation	Conversation guidelines
VC1	Let’s meet	Presentation and data that identify us. Autobiographical summary. Description of students’ current educational-school situation.	First, introduce yourself: What is your name? How old are you? Where were you born? Where do you live? With whom? What is the city you live in like? You can talk about your family, your childhood, your classes: What classes are you taking? What is your favourite? Which one do you like the least? Which one requires the most effort? Which one the least? Why? What are your teachers like? And your classmates?
VC2	Governance of schools	Governing bodies (identification, functions, promotion, etcetera).	How many people make up the management team–e.g. Head teacher, Head of Studies, Secretary–in primary schools in the USA and in Spain? What functions do they have? How are they chosen?
VC3	Organisation of teachers, students and students’ families in schools	The organisation of the teaching staff. Organisation of the student body. Parents’ involvement. Forms of participation. Concretisation of participation. Team work. Students’ rights and duties.	Do teachers in the USA and Spain work as a team? How do you know if they work as a team or not? Give examples of your experiences. For what reasons is a teacher appointed as a counselor in a given class? How are the teaching staff organised in each school? What is the admission policy for the school? Rights and duties of students. What are the criteria for grouping students within each class? Do parents participate in the life of the school? How? Are there family participation structures in the school?
VC4	My hobbies	Management of leisure time. Preferred leisure activities. Planning for future leisure and free time.	Your likes: films, books, sports, meals etc. Your hobbies: What do you do in your free time? What don’t you do but would like to do? Your trips: What did you do last summer? What trips have you done in your life? Which has been the most interesting? Why? What trips do you want to do in the future? Why?
VC5	Attention to diversity in schools	What is diversity? Its implications from the perspective of schools, as well as the individual. Attention to diversity measures. Inclusive education.	What is meant by diversity in your country? What do you understand by diversity in schools? How is diversity managed in schools in your country? Give examples of your experiences. Are there specific programmes for addressing diversity issues in your country? What are these?
VC6	Politics in the USA and Spain	Political parties. Elections. Public opinion.	How many political parties are there? How is the government organised (e.g. Congress, Senate, etc.)? How are presidential elections organised? Did you vote in the last election? Why? What do you think of politics in general?
VC7	Laws relating to education in the USA and Spain	School planning according to legislation. Planning documents at school. School assessment.	What are the official documents that underpin school life? What function does each of them have? How is the assessment of schools carried out in your country? Quality indicators of schools.
VC8	Farewell	Future plans (short, medium and long term). Recapitulation of the experience of the VCs (assessment and possible improvements).	Your plans for the next summer and for the future: What are you going to do this summer? What are you going to do when you graduate from college? Did you like this Skype experience? Why? What would you do differently and why?

VCs 2, 3, 5 and 7 deal with the education systems in primary education in each country. Students had to prepare the topics in advance. The remaining VCs (1, 4, 6 and 8) did not have to be prepared, as their content was of a general nature (Table 1).

### Sample of participants

The educational experience started with 12 couples. Subsequently, due to lack of data, the sample was reduced to 10 couples. The couples shared the same gender and were always the same throughout the educational experience. They were established by taking into account mutually convenient schedules and possible contingencies, in addition to compatibility of tastes and preferences. Although a certain homogeneity of the pairs could be a handicap in terms of intercultural analysis, other factors that could influence the learning objectives (facilitating empathy, avoiding clashes between people in the dyads and facilitating greater communication by sharing tastes and preferences) were also assessed. Therefore, the researchers observed advantages in distributing couples with some homogeneity—with common interests— to facilitate communication.

The sample’s socio–demographic data were: average age (18.8), *SD* of age (2.6); women (70%), men (30%), Caucasian racial origin (91.3%), African American (4.3%), Afro-Caribbean (4.3%).

### ***Data structuring and analysis procedure***

The analysis was split into two phases: (1) establishing the system of categories to be used and (2) analysing the VCs on the basis of the established system of categories.

In phase 1 the researchers constructed a system of categories based on the Affective, Cognitive and Behavioural Dimensions outlined by Chen and Gabrenya (2021), Chen & Young, (2012), Griffith et al. (2016) and Lee and Song (2019) and organised into various sub–dimensions (Table 2). In each of the sub–dimensions, a positive (+) orientation, facilitating effective communication, and a negative (–) orientation, inhibiting communication, were envisaged. The students were coded according to their nationality and gender (e.g., spa/w, usa/m).

**Table 2:** Sub-dimensions, operational definitions and positive tracking indicators (+)

Sub–dimension	Operational definitions and tracking indicators
Behavioural/Att (Beh/Att)	Operational definition: Expressions reflecting a positive attitude during communication. Tracking indicators: Showing a sociable, friendly, courteous, respectful and tolerant attitude. Respecting the rules of politeness and courtesy.
Behavioural/Eff (Beh/Eff)	Operational definition: Expressions of interest in communicating and effort to communicate. Tracking indicators: Attempt to obtain and/or provide further information, make inferences and highlight subtle differences. This is in addition to overcoming and adapting to diverse communication situations.
Affective/Fee (Aff/Fee)	Operational definition: Communications or expressions that reflect ways of feeling (facilitates communication). Tracking indicators: Feeling confident. Overcoming shyness. Expressing oneself sincerely. Being relaxed and receptive.
Affective/Mood (Aff/Moo)	Operational definition: Communications or expressions that reflect a positive mood (facilitating communication). Tracking indicators: Enjoying the interaction. Expressing joy, optimism and/or humour.
Cognitive/Knowledge (Cog/Kno)	Operational definition: Communications which express knowledge. Tracking indicators: Understanding and accepting the culture of others.
Cognitive/Understanding (Cog/Und)	Operational definition: Communications which reflect understanding. Tracking indicators: Understanding cultural differences and similarities. Understanding the other culture. Analysing from an inclusive approach. Changing perspectives in order to understand the other culture. Creating a sense of community without stereotyping.
Cognitive/Learning (Cog/Lea)	Operational definition: Communications demonstrating learning. Tracking indicators: Expressing critical self-perception of their own culture. Establishing common socio-cultural meanings. Learning about differences through comparison. Inferring social norms and networks of interpersonal relationships.

The Affective Dimension includes emotional responses in addition to the management of emotions which may impair the intercultural communicative process. The Cognitive Dimension includes knowledge, understanding and awareness of all cultural and communicative elements which promote effective intercultural communication, including one’s own and that of others. The Behavioural Dimension focuses on the set of verbal and non–verbal skills which show an adaptation of behaviour which promotes effective intercultural communication.

Since the analysis was to be carried out by two researchers, it was necessary to agree on the following: the meaning of a thematic unit, establishing an operational definition for each dimension and sub–dimension, and the specific tracking indicators for each sub–dimension.

Phase 2—the analysis of the communications—consisted of assigning each thematic unit to each sub–dimension, thus converting each fragment of communication into recording units (RU). Throughout this process we worked inductively and deductively. This involved a constant crossover between the theoretical precepts of the research and the communications developed. The transcriptions of the 73 VCs include the spoken word and gestures (nodding acceptance/denial, smiles, etc.). The VCs of two couples were cancelled because they did not have a minimum of 60% of the VCs completed. Nvivo12 software was used to process and structure the information. Content analysis was used as a tool (Krippendorff, 1990, p. 28) and as a research technique (Berelson, 1952).

Inter–coder reliability was performed in order to ensure a degree of conformity and concordance in the assignment of each RU to the given sub–dimensions. Three VCs were randomly selected and a first coding was independently carried out by the two researchers. Following Neuendorf (2002, p. 159), ‘the appropriate sample size should not be less than 50 units’. In rare cases, it needs to be greater than 300 units. In order to analyse inter–coder reliability in this study, we worked with 300 thematic units obtained from 297 interventions. This was done by performing Krippendorff’s alpha. According to Lombard et al. (2002) and Macnamara (2018), Krippendorff’s alpha allows any number of coders, admits nominal variables, considers random agreement and the using of equal marginal proportions for the coders. RUs which were to be assigned to more than one code (i.e., co–occurrences), were not used to obtain Krippendorff’s alpha.

Three rounds of the coding/review were then carried out. In view of the large discrepancies observed in the first coding/review round $\alpha =0.55$, it was decided to revise the operational definitions and tracking indicators. The disagreements were essentially due to different interpretations of the operational definitions (there were overlaps when delimiting attributes and characteristics in each of them). As the RUs were revised, these issues became clearer. In the second round of coding/review, $\alpha =0.66$ was obtained. In a third round, after a further review of the tracking indicators, $\alpha =0.77$ was obtained. Through this iterative process of reiterating the operational definitions among the coders and the subsequent analysis through Krippendorff’s alpha the validation of the Cognitive, Affective and Behavioural Dimension and their sub–dimensions was ensured. The RUs were counted and a percentage frequency index was established for each dimension and sub–dimension. A validation of the results was made by the researchers through re–coding. This validation was carried out with 228 RUs, finding a coincidence between the two rounds of coding of 97.36%. Therefore, it is considered that the assignment of the RUs was correct.

Twelve missing VCs involving several couples could not be analysed, so the mean value (the average of the VCs from the other pairs) was given so as not to distort the results.

## Results

After review process 45,532 RUs were analysed.

### How intercultural communication is distributed considering behavioural, affective and cognitive dimensions?

The results show that the Behavioural Dimension is widely used in communications (Table 3).

**Table 3:** General distribution of dimensions

Dimension	%	Number of RU
Behavioural	61	27,528
Affective	23.8	10,758
Cognitive	15.2	7247

The Behavioural Dimension includes expressions which reflect a positive attitude to communication. This is evidenced by the degree of interest shown by the students in addition to effort, willingness to learn, participate, understand and adapt during communication (e.g., initiating, taking turns, paying attention, showing reciprocity, ending a conversation, etc.). The Affective Dimension deals with intercultural sensitivity, evidenced through positive feelings and emotions. These are communicative expressions which reflect ways of feeling, or states of mind, which facilitate communication (e.g., respect, acceptance, empathy, curiosity, tolerance, resilience, etc.). The Cognitive Dimension refers to communications characterising both intercultural awareness and self-awareness through understanding, cognition and learning about the other culture (e.g., recognising, integrating and applying norms, hierarchies and social-cultural networks, adopting a perspective using cultural information, etc.).

In the independent analysis of each dimension, Table 4 (Behavioural Dimension), 5 (Affective Dimension) and 6 (Cognitive Dimension), show an asymmetric distribution between the different sub-dimensions.

**Table 4:** Distribution of RUs in Affective Behavioural Sub–dimensions

	Of the total (%)	On Behavioural Dimension (%)
Beh/Att–	—	—
Beh/Att+	13.3	21.8
Beh/Eff–	0.1	0.2
Beh/Eff+	47.4	77.8

The following excerpt exemplify communication related to the Behavioural Dimension:

Couple 3–VC6 spa/w: *Of course, don’t worry. If you know, you tell me, and if you don’t, nothing happens. Don’t worry, I’ll explain it to you*. [Beh/Eff+]

usa/w: Yes.

spa/w: *Let’s learn a little bit and that’s it. Do you want me to tell you a little bit about what it’s like in Spain?* [Beh/Eff+]

usa/w: Yes.

Positive behaviour with the purpose of allowing communication to develop, is evident in the RUs (in italics). Expressions that show interest, effort, willingness to learn, participate, understand and adapt during communication are in italics (Table 5).

**Table 5:** Distribution of RUs in the Affective Sub–dimensions

	Of the total (%)	On Affective Dimension (%)
Aff/Moo–	2.3	9.8
Aff/Moo+	9.7	40.6
Aff/Fee–	0.1	0.4
Aff/Fee+	11.9	50.2

Excerpt which exemplify the Affective Dimension include the following:

Couple7–VC8 spa/w: And what was I going to say, ah, did you like the Skype experience?

spa/w: *I’m also enjoyed talking to you these days* [Aff/Fee+], *because I’ve felt very comfortable* [Aff/Fee+] *and I like your way of thinking.* [Aff/Fee+]

The examples shown above highlight expressions of respect, acceptance, empathy and tolerance denoting positive feelings and emotions (intercultural sensitivity) and facilitating intercultural communication.

In the Cognitive Dimension the sub–dimensions observed are as shown in Table 6.

**Table 6:** Distribution of RUs in the Cognitive Sub–dimensions

	Of the total (%)	On Cognitive Dimension (%)
Cog/Lea–	0. 2	1. 3
Cog_Lea+	6. 8	44. 5
Cog/Und–	2. 5	16. 6
Cog/Und+	2. 2	14. 6
Cog/Kno–	3. 1	20. 7
Cog/Kno+	1. 1	7. 4

The following is an example regarding the Cognitive Dimension:

Couple 9–VC5b spa/w: *So children are learning, they are playing, but they don’t realise that they are learning at the same time. Thus, I think that the iPads article in your country that you told me about is very interesting, because children are playing, but in reality they are learning.* [Cog/Lea+]

usa/w: Yes.

spa/w: *Then I think new technologies are super important.* [Cog/Lea+]

usa/w: *And it is the way to make it fun, for learning new things and for many students.* [Cog/Lea+]

The RUs shown above demonstrate intercultural understanding and learning. They also convey a sense of gaining perspective of a participant’s personal context through information from the participant of the other culture. Thus, new information is recognised and integrated and even new inferences are made.

### What typologies of intercultural educational online communications are observed among university peers?

A one–way between–groups multivariate analysis of variance was performed to investigate typologies of topics (generic/specific) in different dimensions on intercultural communicative competence through videoconferences in Higher Education. Three dependent variables were used: Behavioural, Affective and Cognitive Dimension. The independent variable was typology. Preliminary assumption testing was conducted to check with no serious violations noted (Pallant, 2007; Tabachnick & Fidell, 2018):

- Normality: Aff y Beh ($$p>.05)$$, Cog ($$p<.05)$$
- Univariate and multivariate outliers: Aff, 2; Beh, 1.
- Homogeneity of variance–covariance matrices: ($$p>.05)$$
- Multivariate normality: Mahalanobis distance, one case (ID = 24) exceeds the critical value of 16.27.
- Homogeneity of variances: Aff, Cog and Beh ($$p>.05)$$
- Multicollinearity: correlation, Aff/Beh ($$r=.72, p=.00)$$, Aff/Cog $$r=.52, p=.00)$$, Beh/Cog $$(r=.67, p=.00)$$

$\left(r=.67,p=.00\right)$. A high correlation between the three dimensions has been found, however up to $r=.8$ is reasonable to accept for the MANOVA test (Pallant, p. 282).

**Fig. 3:**
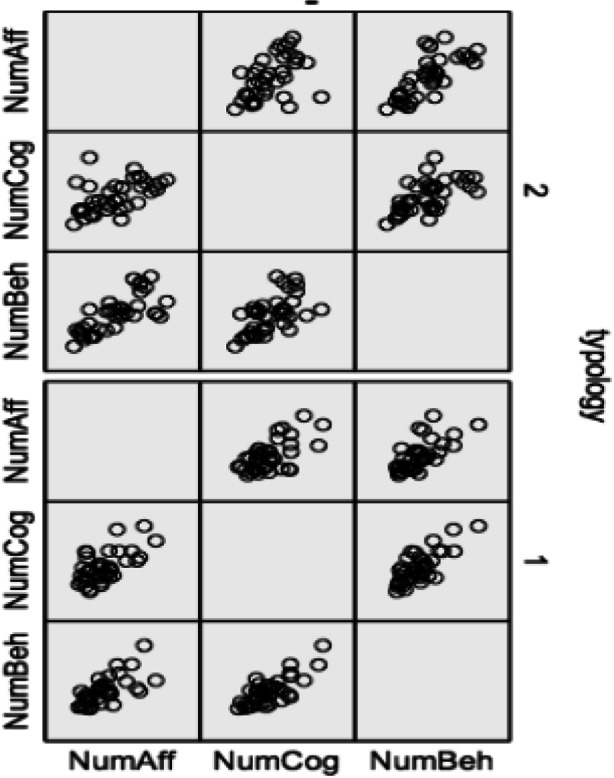
Scatterplot. Typology (1: generic; 2: specific) and Affective, Cognitive, Behavioural Dimensions.

- Linearity: scatterplot (Fig. 3).

There was a statistically significant difference between typologies of topics on the combined dependent variables, $F \left(\text{3,76}\right)=6.32, p=.001$, Wilks’ Lambda = 0.80; partial eta squared = 0.20, following Tabachnick and Fidell (2018, p. 55) is small size. When the results for the dependent variables were considered separately, the only difference to reach statistical significance, using a Bonferroni adjusted alpha level of 0.017, was Affective Dimension $F \left(\text{1,78}\right)=3.63, p=.001$, partial eta square = 0.13. A follow-up inspection of the mean scores indicated that Affective Dimension reported a statistically significative difference between generic topic VCs $\left(M=162, SD=86\right)$ and specific topic VCs $(M=106, SD=60)$.

### How do intercultural educational online communications develop over time?

One–way repeated measures ANOVAs were conducted to observe if there are significant differences in the development of Behavioural, Affective and Cognitive Dimensions of intercultural online communications over time (VCE1 to VCE8). Preliminary assumption testing was conducted (Table 7), with no serious violations noted. The mean and standard deviation are presented in Table 8.

**Table 7:** Preliminary assumption testing

	Behavioural	Affective	Cognitive
Homogeneity of variances. Levene’s test	*p* > .05	*p* > .05	*p* > .05
W Mauchly’s test of sphericity	0.003 (*p* = .14)	0.001 (*p* = .04)	0.015 (*p *= .56)
Normality. Shapiro-Wilk	*p *> .05, except VC6	*p* > .05, except VC6	*p* > .05, except VC6
Outliers. Boxplots	3 (VC2), 3 (VC6), 1 (VC7)	1 (VC6), 2 (VC8)	1 (VC4), 1 (VC6), 1 (VC7)

**Table 8:** Descriptive statistics for Behavioural, Affective and Cognitive Dimension with statistics test scores for VC1 to VC8

Time period	Behavioural				Affective				Cognitive		
	*N*	*M*	*SD*		*N*	*M*	*SD*		*N*	*M*	*SD*
VC1	10	387.90	171.12		10	170	92.66		10	84.40	31.42
VC2	10	367.60	140.81		10	123.90	53.22		10	109.20	34.14
VC3	10	299.70	179.93		10	83.00	68.61		10	85.00	40.12
VC4	10	354.70	162.17		10	140.70	70.61		10	80.30	42.69
VC5	10	302.80	129.62		10	119.80	63.67		10	84.00	32.51
VC6	10	428.60	189.04		10	147.90	81.36		10	92.10	36.13
VC7	10	276.10	159.14		10	97.50	54.22		10	70.60	35.04
VC8	10	340.30	118.43		10	189.70	105.57		10	83.80	36.46

In the Behavioural Dimension, Mauchly’s test indicates that there is a violation of the assumption of sphericity $${(\chi }^{2}\left(27\right)=37.15, p=.14)$$, therefore, the degrees of freedom have been corrected with the Huynh–Feldt sphericity (Huynh & Feldt, 1976) estimate due to the sample is small $$\left(\epsilon =0.74\right)$$. There was significant effect for time, Wilks’ Lambda = 0.03, $$F\left(\text{7,3}\right)=14.37, p=.02$$, multivariate partial eta squared = 0.97. According to the results obtained in pairwise comparisons, there are no statistically significant differences in the behavioural dimension ($$p>.05)$$ except in VCE6/VCE7 $$(p=.05)$$. The effect size is very high *(*$$p>.05$$, partial eta squared = 0.97). The difference between groups in terms of standard deviation units is Cohen’s *d* = 0.87. The mean and *SD* of these VCs are shown in Table 8.

In the Affective Dimension, the Mauchly’s test indicates that there is a violation of the assumption of sphericity $({\chi }^{2}\left(27\right)=43.57, p=.04$, therefore the degrees of freedom have been corrected with the Huynt–Feldt estimate of sphericity due the sample is small $(\epsilon =0.64)$. There was a significant effect over time, Wilks’ Lambda = 0.05, $F\left(\text{7,3}\right)=8.38, p=.05$, multivariate partial eta squared = 0.95.

Regarding pairwise comparisons, there are no statistically significant differences over time in the Affective Dimension ($$p>.05)$$ except for VCE03 (specific topic: Organisation of teachers, students and students’ families in schools) with VC1, VC4 and VC8, with a very large effect size $$(p>.05)$$, partial eta squared = 0.95). The difference between groups in terms of standard deviation units is Cohen’s *d* = 1.06 (VC3–VC1), Cohen’s *d* = 0.82 (VC3–VC4) and Cohen’s *d* = 1.19 (VC3–VC8).

In Cognitive Dimension, Mauchly’s test shows that assumption of sphericity is not violated $\left({\chi }^{2}\left(27\right)=0.01, p=56\right)$. No significant effects over time were observed (Wilks’ Lambda = 0.14, $F\left(\text{7,3}\right)=2.6, p=.23)$. This statement is also supported by the results of pairwise comparisons where $p>.05$ is observed.

**Fig. 4:**
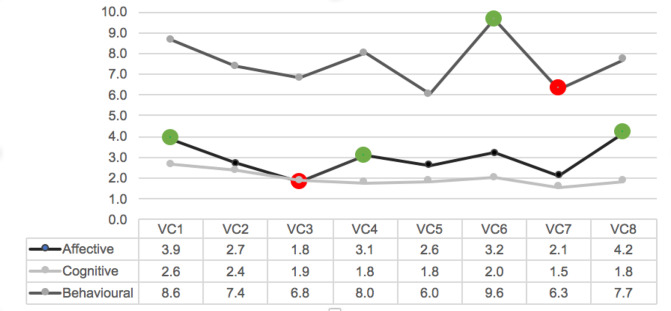
Evolution of communications over time. Specific topic (red), generic topic (green). 45,532 RU = 100%.

Figure 4 shows that generic topics are the most frequently discussed in Affective and Behavioural dimensions communications (VC8: Farewell, VC6: Politics in the USA and Spain). However, the VCs whose contents deal with specific educational aspects (red) have a lower incidence (VC3: Organization of teachers, Students and students’ families in schools; VC7: Educational laws in the USA and Spain).

**Fig. 5:**
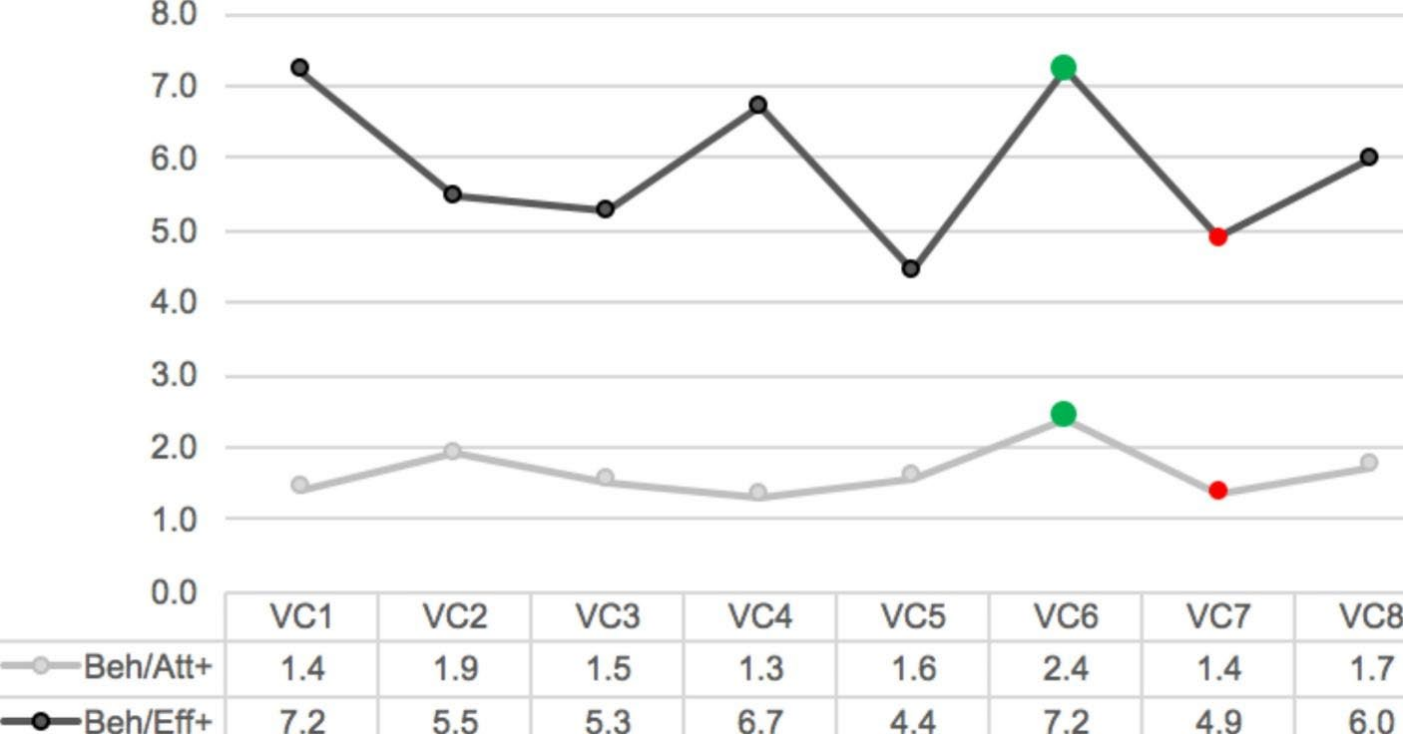
Percentage of communications of the Behavioural Sub–dimensions over time. Specific topic (red), generic topic (green). 45,532 RU = 100%.

Figure 5 shows the RUs that are linked to attitude and effort. Neither sub–dimension is represented in the communications in a negative way. Communications with positive content we found, in particular, expressions of interest and effort to initiate or keep communication going.

However, it could be due to the fact that they enter into personal opinions on issues with ideological and topical content (VC6, politic). In the case of Beh/Att+, participants use expressions (postscripts, affirmations, restatements, reiterations and so on) which, mostly, allow the continuity of their partner’s discourse. They also use other expressions that show facilitating behaviours. All this enriches the communicative exchange (kindness, politeness, courtesy and respect). In relation to Beh/Eff + these expressions were recorded when new lines of argument, conversation, enquiry and deepening that exceed from the main topic of the VC were introduced.

**Fig. 6:**
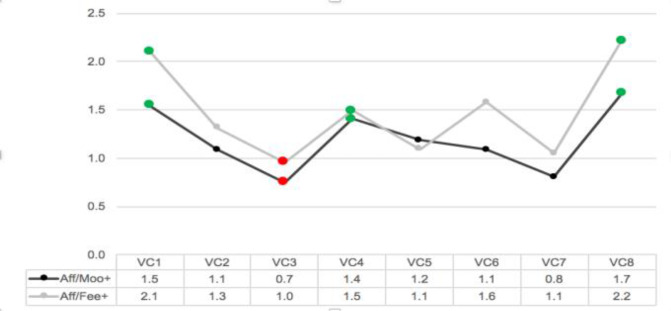
Percentage of communications of the Affective Sub–dimensions over time. Specific topic (red), generic topic (green). 45,532 RU = 100%.

On the other hand, in the Affective Dimension (Fig. 6) Aff/Fee + is the Sub-dimension with the highest number of RUs, reflecting the prevalence of positive moods which facilitate intercultural communication. Its highest records are observed in those VCs that developed generic topics, although with prominence at the beginning and at the end of the educational experience (VC1, let’s meet, and VC8, farewell). However, a large part of the Aff/Fee + is registered in relation to trust, openness and sincerity between participants, while the Aff/Moo + register expressions of optimism, good mood, joy and humour.

In the Cognitive Dimension, the most prolific RUs are those that refer to own culture critical self–perception, or those which establish common meanings, learning from each other’s differences and inferring social norms (Cog/Lea+).

**Fig. 7:**
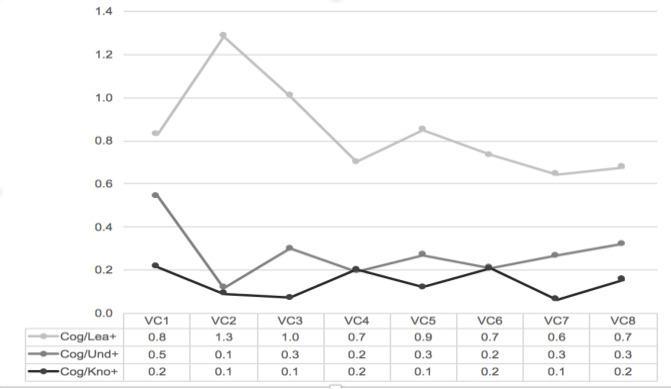
Percentage of communication of Cognitive Sub–dimensions over time (45,532 RU = 100%).

The ANOVA test indicated that wasn’t significantly different from VC1 to VC8, however, we observe in Fig. 7 that the largest number of RUs in Cog/Lea + is produced in two VCs that develop specific themes (VC1 and VC3). However, they were also registered when complementary resources were used in order to move the communication forward (second language, use of technology, lateral examples, comparatives).

In the communications analysed, the Cog/Kno + Sub–dimension was used by Spanish students when information or data relating to where the partner comes from was known, remembered or used. It was also used when a word (in the partner’s language), or a piece of information, was integrated into the dialogue in order to improve communication. Cog/Und + was used when a cultural element, information, fact or concept was understood, and this understanding was explained or when an explanation, reiteration or answer in the form of a question was requested (mainly with regard to the Americans).

## Conclusions

This paper analyses online communications between pairs of culturally diverse university students. The analysis focuses on the development of Behavioural, Affective and Cognitive Dimensions, and their sub–dimensions, with the aim of exploring the typology and development of their communications.

Our research has found, as Deardorff (2020) and Heggernes (2021) point out, a respectful and dialogue-based intercultural communication. On the other hand, in this study it has been observed, in line with Griffith et al. (2016), that ICC is an interactive process and not so much a static construct. Fluent communication was found and no difficulties in cultural exchanges through videoconferencing were observed. Contrary to what Van der Zwaard & Bannink (2019) argue, in this research no communications were found concerning the barrier produced by having to respond quickly in this online environment.

The most frequently found communications are related to the Behavioural Dimension, the present study finds expressions that show a positive attitude towards communication, as well as interest in and an effort to maintain it. Participants try to obtain, or provide, information which enables smooth communication. They also make deductions, highlight subtle differences and overcome different communication scenarios. The large number of URs registered relating to Beh/Eff + demonstrate the ability to use the verbal, and non–verbal, behaviours of their partner as noted by Chen and Young (2012). In addition, both partners demonstrate the ability to initiate a conversation, take turns in speaking and finally end the conversation. The participants’ interest in developing the communication is observed when information that has been given goes beyond to a closed answer or to the VC script, when a deeper explanation has been requested and when complementary resources have been used (using the second language, given examples, …) to clarify the information given. The use of communication strategies, and the ability to take an interest in the partner’s personal and cultural identity, are also evident. Other actions found were, when participants were attentive to the conversation thread allowing the partner’s discourse continuity, also using expressions of social competence (kindness, courtesy, politeness, politeness rules, respect). The negative communications registered are anecdotal, which shows the positive predisposition of the participants to keep communication going.

This study found that Affective Dimension reflects expressions of positive emotions and ways of feeling. Here the communications demonstrate trust between peers, showing them as both relaxed and receptive. In addition, they enjoy their interaction by expressing joy, humour or optimism, although to a lesser extent. As in Toscu and Erten’s study (2020), a clear enthusiasm for learning about other cultures was found, showing a great deal of affective communication, including displays of interest and curiosity. They also showed signs of closeness and trust, in line with the studies of Dai (2019) and Lenkaitis et al. (2019). Furthermore, communications showing a marked open–mindedness in relation to the other culture can be observed as well as a respect for the differences participants find with respect to their own culture. When faced with ambiguity, tolerance is also seen in all the VCs, as well as ways of overcoming difficulties, whether due to discomfort, confusion or anxiety. Therefore, the educational experience, in our case the VCs, has become a way of creating social relationships between participants, as held by Griffith et al. (2016). However, in a very small percentage, there are negative records in this dimension. Aff/Moo– is found in communications relating to the present time, when pessimism, discouragement, sadness or a sense of failure is expressed about a particular issue or current situation. Negative feelings are expressed when technical problems arise, and these are seen to bias the communication, in line with the conclusion of Custer (2017). These problems were related to the internet connection. According to Godwin–Jones (2019) this can lead to ‘devoting all their [students’] attention to the technology itself, at the expense of a deeper negotiation of social and cultural meanings, let alone worldviews’ (p. 175). However, it was not observed that these technical problems had an impact on the development of the VCs, beyond minor losses of time and a small increase in RUs related to Aff/Moo–.

Regarding to Cognitive Dimension, communications pertaining to intercultural knowledge, reflecting understanding of the other culture or demonstrating intercultural learning, is the dimension least frequently encountered. Here, communication demonstrating learning is seen to be the most conspicuous (for example critical self–perception, establishing common socio–cultural meanings, inferring social norms). However, in the negative dimension (for example displays of ignorance and lack of understanding of the other culture), we find communications which could be a barrier to communication. In line with Cheng and Joung (2012), a certain ability to infer social norms, the non–use of personal biases or stereotypes was observed although, in some cases, generalisations were found. In addition, some communications were found to refer to a partner’s historical and geopolitical background. Further inferences are possible just by comparing the Cognitive Dimension with the Behavioural and Affective Dimensions. This is because the negative Cognitive Sub–dimensions (although significantly fewer in number than the positive ones) have reached a higher level compared with the other dimensions. It can be seen how, in all VCs, in Cog/Kno y Cog/Und the number of RUs the negative sub–dimension higher than positive sub–dimension. This is proof of the difficulty of videoconferencing when there is a lack of cultural information and knowledge of the partner’s language.

In this study, differences were found in the generic/specific communications related to the Affective Dimension. This agree with the results of Eren (2021) and Godwin–Jones (2019), in that ‘safe’ topics have proven to be useful in increasing communications as opposed to specific topics. A higher number of RUs has been observed in those VCs where generic topics are addressed, and a lower number of them in those developing specific educational topics. In this way, we can see how the greater or lesser complexity and specificity of the topic is capable of inhibiting or enhancing communication between videoconferencing participants in intercultural environments. In the Affective Dimension, where generic topics enhance communication, while educational topics inhibit it.

The suggestion in previous studies that ICC can be enhanced by online intercultural experience (Akdere et al., 2021; Lee & Song, 2019) has not been fully demonstrated in our study. An analysis of the communications over time indicates that the topic addressed is a factor with implications for the development of Behavioural and Affective Dimensions but this is not happening in Cognitive Dimension.

In the Behavioural Dimension, a statistically significant evolution has been found between VC6 (generic topic) and VC7 (specific topic), which are the highest and lowest UR found in this dimension. According to this study, expressing opinions related to politic makes possible a higher level of development of the conversation allowing, especially, opening new lines of conversation, inquiring about opinions or asking explanations and, to a lesser extent, giving continuity to the partner’s discourse, which shows effort, involvement and interest. On the other hand, conversation on a very specific topic (education laws in both countries), which students needed to have prepared in advance, did not arouse their interest in developing the Behavioural Dimension, but rather they observed a compliance with the conversation script in order to end the conversation quickly. There was a high degree of trust between videoconferencing participants, also over time progression was found on Affective Dimension. In line with the proposals of Griffith et al. (2016), close relationships forged with people from different cultural backgrounds demonstrate cultural self–efficacy. Through the analysis over time, it has been observed that VC3 (specific topic, with the lowest mean of its dimension) has statistically significant differences with three previous and subsequent VCs with generic topic (VC1, VC4 and VC8). The topic of VC3 (Organization of the teaching staff, families and student, participation, rights and duties) did not facilitate the development of the Affective Dimension in neither of the two components analysed (Aff/Moo + and Aff/Fee+: confidence, openness in responding, truthfulness, freedom to respond, optimism in conversation, motivation to the partner, expressions of enjoyment, positive wishes to the partner). However, these expressions were especially found at the beginning and at the end of the educational experience. This situation can be explained in VC1 (presentation, description of students’ situation and first contact through VC) by the high expectations that the students had at the beginning of the experience, and in VC8 (future plans, recapitulation of the experience) by the fact that it was an enjoyable experience for them. Also, in VC8 the high rate of affective elements can be explained by the fact that the couples know each other and, over time, a climate of trust has been created.

Thus, the eminently social character of these topics and the fact that VC participants do not have to respond to the requirements of an educational topics seem to reduce the complexity of communication and to develop learning, understanding and knowledge processes. On the other hand, a significant difference was also found between VC3 and VC4 (My hobbies). The topic, in this case, has also been able to establish the incidence of the Affective Dimension. In the analysis of the Affective Dimension over time, it is observed that sub–dimensions Aff/Fee + and Aff/Moo + increase the number of RUs in those VC that develop generic topics with a social and affective character. Such regularity suggests a relationship between communications involving positive emotions although, in some cases, there is no statistically significant difference. The appearance of a positive emotion on the part of one participant gives rise to the appearance of another positive emotion in his or her partner.

In the analysis over time of the Cognitive Dimension no statistically significant differences were found between the VCs. Furthermore, contrary to the study of Eren (2021), no increase in the critical intercultural development of the participants was observed. Neither a reinforcement nor a decrease in negative stereotypes was found. Therefore, this study cannot conclude anything in relation to the study by Flowers et al. (2019), which reports reinforcement, nor to the study by Eren (2021), which reports a decrease in negative stereotypes. On the other hand, Cog/Lea + appears as the sub–dimension with the most records, especially in those VCs with educational topics. It is possible to infer that, when it comes to VCs with educational topics, the expression of personal opinion about what has been discussed, the expression of a critical self–perception about what has been discussed, comparisons with the partner’s context and the expression of common meanings are facilitated.

## Limitations, implications and future perspective

As for the limitations of the study, we point to the distribution of the sample regarding gender (women, 70%; men, 30%). Another limitation has to do with the selection of the sample of subjects, since it is not based on statistical criteria that are representative of the population, but rather seeks to combine minimum heterogeneity within a framework of homogeneity. In this way, our intention was to establish certain common and relevant attributes (gender, tastes and preferences). However, this may have had an impact on the RU records, especially in the lower proportion of negative communications in relation to positive ones, since greater homogeneity in the sample could inhibit the emergence of negative communications and enhance positive ones. Nevertheless, in the Cognitive Dimension a greater number of negative communication RUs was observed, so it could be that homogeneous pairing had a minor influence on this dimension. In addition, 50% of our sample experienced a language barrier, as the VCs were developed with an L2, which might have contributed to misinterpreting answers. Finally, this research may find a limitation in its validity and reliability because of its reliance on content analysis for data extraction. A careful procedure has been followed to avoid, as far as possible, this situation.

Our findings have implications which should develop a more internationalised, and interculturally sensitive, environment for higher education institutions. The study also has implications for teachers because it highlights the implementation of useful, technology–based methodologies for the development of ICC. Finally, in our opinion, students would benefit from a fully functional methodology in order to practice intercultural competencies in a technified and globalised world.

As no statistically significant differences were found in the overtime evolution of the Cognitive Dimension, the authors consider the possibility that this may be due to the experience instructional design. For instance, a greater monitoring by the teachers (more exhaustive conversation guide, intermediate seminars, etc.) could have facilitated the development of this dimension. The answer to this question we purpose as a prospect for future research. Taking into account on the one hand, Lee and Song’s (2019) view that the Behavioural Dimension is the least studied with regard to ICC and, on the other, that this dimension is the most prominent in the current research, the authors believe that a future line of enquiry should focus on Behavioural Dimension and thus contribute to the further study of online intercultural communication.

## Data Availability

This study is guided by the Code of Good Practice in Research of University of Granada, Spain. Vice-Rectorate for Research and Knowledge Transfer.
